# Accelerated Breathing

**Published:** 1884-03

**Authors:** 


ACCELERATION OF THE BREATHING is never absent in any case of actual consumption. In the last few weeks of life a few steps puts the patient out of breath, even if those steps be over a level floor. But long before this there was observed an inability to walk fast without considerable discomfort. In fact, a slow and measured tread is the symptom which first strikes the ordinary observer. The man himself may be scarcely an apparent invalid, except on close scrutiny. He may be lively in conversation, he may eat heartily, may have little or no cough, but any effort on your part to induce him to greater bodily activity is instinctively avoided. At a still earlier period one thing has been forced upon the attention of the patient: that he does not mount a pair of stairs with the same celerity as formerly. In days long ago he could take two or three steps at a stride, and even feel the better for it when he reached the top; but now, such an effort would make him puff and blow inconveniently. At an earlier period still, there is an observable feeling of tiredness about the legs and knees on going up stairs, a feeling of weakness there, not known in earlier years, implying a want of bodily vigor not pertinent to that stage of life.

It is to be hoped that no one will haste away with the impression that a little feeling of fatigue in going up a pair of stairs is a sign of consumption. This book is not written for quibbling critics; it is written for the instruction of people of sober views, who can look at a subject steadily, willing to be informed, but unwilling to run away with either end of the subject, or precipitate themselves into the weakness of extremes

But it is an instructive fact, that if this easiness of fatigue in ascents, be conjoined with the quick pulse, and be so for months in succession, it is an impressive warning of coming consumption, and millions would be saved, if it were heeded.

A man may be lazy, it may be summer time, or various other things may give rise to a transient exhibition of acceleration in pulse and breath, or they may arise from the mere habit of sedentariness; but there is one easy, decisive, infallable method of determining whether these symptoms are from transient causes, or from an actual change going on in the structure of the lungs themselves; and that is, by measuring the quantity of air which the lungs are capable of drawing in, at one deep, full, free breath, that is done by the use of an instrument often seen in the street, "The Lung Measurer," or "Spirometer," it has not found favor here, as it is expensive, is liable to abuse, and to the mass of physicians, the opportunities of making varied observations upon it, are not offered. Besides, it requires time and patience to classify the phenomena which it presents; and unless a man have a considerable practice of that kind, it does not pay, either in money or in data for scientific results.

In the Author's practice, then, the great preponderating indications of consumption are, accelerated pulse and breathing; no judicious practitioner will rely wholly on the pulse, or any other two or three symptoms, but on the whole set of symptoms which any given case presents, together with the history of his life, his temperament, his habits, his hereditary tendencies and idiosyncracies, that is, peculiarities, of constitution.

The more obvious symptoms of consumption have been already sketched in a general way. So few persons recover from what is called confirmed consumption, that it was not considered profitable to enter into a critical enunciation and description of all the symptoms, real or imaginary, and in their various stages, degrees and progress; such a thing would materially detract from the practical, utilitarian design, which has been ever prominent. There is so little hope of clearing out the Augean stable of a confirmed consumptive, in any given case, that it is considered only worth while to direct attention critically to the symptoms and stages which admit of a comparatively speedy and permanent arrest or cure.

The large majority of deaths by consumption, are out of married life, indicating the general fact, that its victims are mainly the young, from twenty to thirty.

